# Detecting rare gene transfer events in bacterial populations

**DOI:** 10.3389/fmicb.2013.00415

**Published:** 2014-01-07

**Authors:** Kaare M. Nielsen, Thomas Bøhn, Jeffrey P. Townsend

**Affiliations:** ^1^Department of Pharmacy, Faculty of Health Sciences, University of TromsøTromsø, Norway; ^2^GenØk-Centre for Biosafety, The Science ParkTromsø, Norway; ^3^Department of Biostatistics, Yale UniversityNew Haven, CT, USA; ^4^Program in Computational Biology and Bioinformatics, Yale UniversityNew Haven, CT, USA; ^5^Program in Microbiology, Yale UniversityNew Haven, CT, USA

**Keywords:** lateral or horizontal gene transfer, DNA uptake, modeling, monitoring, sampling, antibiotic resistance, GMO, biosafety

## Abstract

Horizontal gene transfer (HGT) enables bacteria to access, share, and recombine genetic variation, resulting in genetic diversity that cannot be obtained through mutational processes alone. In most cases, the observation of evolutionary successful HGT events relies on the outcome of initially rare events that lead to novel functions in the new host, and that exhibit a positive effect on host fitness. Conversely, the large majority of HGT events occurring in bacterial populations will go undetected due to lack of replication success of transformants. Moreover, other HGT events that would be highly beneficial to new hosts can fail to ensue due to lack of physical proximity to the donor organism, lack of a suitable gene transfer mechanism, genetic compatibility, and stochasticity in tempo-spatial occurrence. Experimental attempts to detect HGT events in bacterial populations have typically focused on the transformed cells or their immediate offspring. However, rare HGT events occurring in large and structured populations are unlikely to reach relative population sizes that will allow their immediate identification; the exception being the unusually strong positive selection conferred by antibiotics. Most HGT events are not expected to alter the likelihood of host survival to such an extreme extent, and will confer only minor changes in host fitness. Due to the large population sizes of bacteria and the time scales involved, the process and outcome of HGT are often not amenable to experimental investigation. Population genetic modeling of the growth dynamics of bacteria with differing HGT rates and resulting fitness changes is therefore necessary to guide sampling design and predict realistic time frames for detection of HGT, as it occurs in laboratory or natural settings. Here we review the key population genetic parameters, consider their complexity and highlight knowledge gaps for further research.

## Introduction to HGT in bacterial populations

Bacteria in natural populations are known to import and integrate exogenous genetic material of diverse, often unidentified, origins (Eisen, 2000; Lawrence, 2002; Nakamura et al., 2004; Chen et al., 2005; Didelot and Maiden, 2010). Bacterial genomes can be exposed not only to the multitude of sources of exogenous DNA present in their natural environments (Levy-Booth et al., 2007; Nielsen et al., 2007; Pontiroli et al., 2007; Pietramellara et al., 2009; Rizzi et al., 2012), but also to introduced sources of novel DNA such as the fraction of recombinant DNA present in genetically modified organisms (GMOs) (Nielsen et al., 2005, 2007). DNA exposure can potentially lead to horizontal gene transfer (HGT) events dependent on the multitude of parameters that govern HGT processes in various environments (Dubnau, 1999; Bensasson et al., 2004; Thomas and Nielsen, 2005; Popa and Dagan, 2011; Domingues et al., 2012a; Seitz and Blokesch, 2013).

For long-term persistence of infrequently acquired genetic material in new bacterial hosts, a conferred selective advantage is considered necessary (Feil and Spratt, 2001; Berg and Kurland, 2002; Pettersen et al., 2005; Johnsen et al., 2009; Kuo and Ochman, 2010). Experimental investigations have shown that most HGT events that integrate into the bacterial chromosome are deleterious (Elena et al., 1998; Remold and Lenski, 2004; Starikova et al., 2012). Thus, in terms of the persistence of its signature and its effects on fitness, HGT processes resemble routine mutational processes that take place at similarly low frequencies in bacteria and that are eventually lost from the population due to lack of a conferred advantage (Kimura and Ohta, 1969; Jorgensen and Kurland, 1987; Lawrence et al., 2001; Mira et al., 2001; Koonin and Wolf, 2008; Johnsen et al., 2011). However, the larger size range of DNA transferable through single HGT events increases the potential for rapid acquisition of functional traits in bacteria when compared to single mutational events (Overballe-Petersen et al., 2013).

Both HGT and mutation should be seen as processes that occur continuously in bacterial populations. For HGT, the combinatorial possibilities are nearly unlimited as the substrates are diverse evolving DNA sequences. Due to a number of barriers and limitations to HGT, only a few of these combinatorial possibilities will materialize at a given time. When individual cells with rare HGT events (and or mutations) are positively selected under particular conditions, they may become important sources of bacterial population adaptation and evolution (Imhof and Schlötterer, 2001; Townsend et al., 2003; Orr, 2005; Barrett et al., 2006; Sousa and Hey, 2013). Different bacterial species and strains are likely to experience variable contribution of HGT to their evolutionary trajectories (Spratt et al., 2001). For instance, positive selection of bacteria after HGT of drug resistance determinants plays a central role in the evolution of resistance to antibacterial agents (Bergstrom et al., 2000; Heinemann and Traavik, 2004; Aminov and Mackie, 2007; Aminov, 2010, 2011).

The detection of HGT events in a given bacterial genome can be performed retrospectively through bioinformatics-based comparative analyses (Spratt et al., 2001; Nakamura et al., 2004; Didelot and Maiden, 2010; Didelot et al., 2010). Alternatively, events may be detected via focused experimental efforts on defined bacterial populations under controlled conditions in the laboratory (Nielsen et al., 1997, 2000), or monitoring efforts on subsamples taken from bacterial populations present in various environments, e.g., from soil, water, wounds, or gastrointestinal tracts (Nielsen and Townsend, 2004; Pontiroli et al., 2009; Aminov, 2011). The latter monitoring approach has limitations, but may enable the identification of HGT events as they occur in the context of complex interactions in and between diverse bacterial communities.

Representative analysis of HGT events in bacterial communities depends on knowledge of the structure and population dynamics of the study population and the sequence of the DNA transferred. Detection strategies nevertheless frequently rely on hidden or implicit assumptions regarding the distribution and proportion of the individual cells in the sampled larger bacterial population that would carry the transferred DNA sequences (Nielsen and Townsend, 2004; Heinemann et al., 2011).

Risk assessments of genetically modified (GM) organisms also consider the potential for HGT of recombinant DNA inserts (Nielsen et al., 2005; EFSA, 2009). For instance, the large-scale cultivation of GM-plants, i.e., on about 170 million hectares worldwide (James, 2012), results in multitudinous opportunities for bacterial exposure to **recombinant DNA** and therefore, opportunities for unintended horizontal dissemination of **transgenes** (EFSA, 2004, 2009; Nielsen et al., 2005; Levy-Booth et al., 2007; Wögerbauer, 2007; Pietramellara et al., 2009; Brigulla and Wackernagel, 2010). In laboratory settings, experimental studies have demonstrated that single bacterial species can take up and recombine with DNA fragments from GM-plants under optimized conditions (e.g., Gebhard and Smalla, 1998; de Vries et al., 2001; Kay et al., 2002; Ceccherini et al., 2003). In natural settings, negative or inconclusive evidence for HGT has been reported from most sampling-based studies of agricultural soils, run-off water and gastrointestinal tract contents (Gebhard and Smalla, 1999; Netherwood et al., 2004; Mohr and Tebbe, 2007; Demanèche et al., 2008; Douville et al., 2009).

KEY CONCEPT 1 | Recombinant DNA DNA that has been recombined in the laboratory using *in vitro* methods, and then transferred into the genome of transgenic organism via various gene transfer techniques. Such DNA usually originate from several different species.

KEY CONCEPT 2 | Transgene Functional unit of recombined DNA present in the genome of a recombinant organism (also called genetically modified or transgenic organism). The word transgene is sometimes used synonymously with “insert” or “inserted DNA.”

Between idealized laboratory conditions and investigations of complex ecosystems, HGT research suffers from significant methodological limitations, model uncertainty, and knowledge gaps. Most research on HGT from GM-plants to bacteria has been performed via bacterial screening after a limited time period following transgene exposure, perhaps in part because only limited explicit considerations of the population dynamics of HGT events have been available to guide sampling design and data analysis (Heinemann and Traavik, 2004; Nielsen and Townsend, 2004; Nielsen et al., 2005; Townsend et al., 2012).

Given the generally low mechanistic probability of horizontal transfer of non-mobile DNA in complex environments such as soil or the gastrointestinal tract, HGT events will initially be present at an exceedingly low frequency in the overall bacterial population. It may therefore take months, years, or even longer for the few initially transformed cells to divide and numerically out-compete non-transformed members of the population to reach proportions that can be efficiently detected by a particular sampling design (Nielsen and Townsend, 2004). The generation time of bacterial populations that are potential recipients for HGT events is therefore of high importance for the determination of sample size and choice of detection methods.

**Figure 1:**
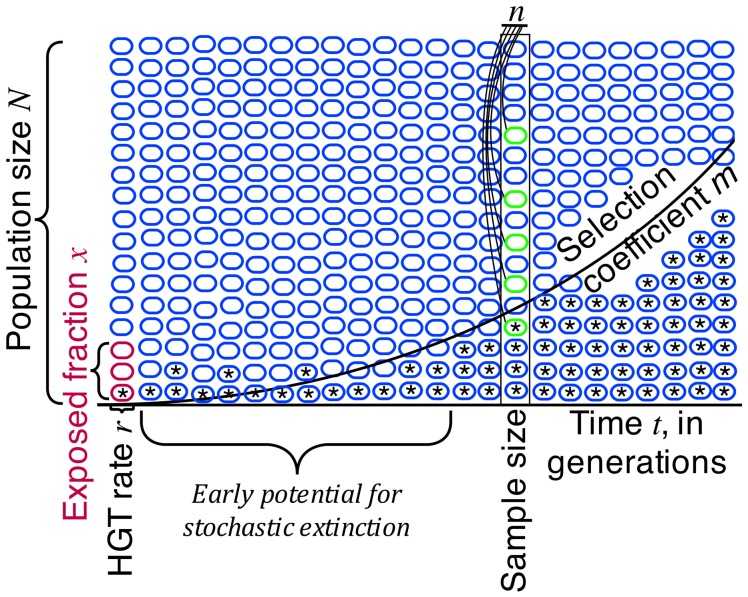
**Population genetic modeling of HGT suggests several key quantities are important to designing any sampling-based assay of horizontal gene transfer (HGT) in large populations.** The HGT rate *r* and the exposed fraction *X* play significant but ultimately minor roles in the population dynamics, most likely impacting only the number of original opportunities for horizontal spread of genetic material. The malthusian selection coefficient *m* of the transferred genetic material and the time in recipient generations *t* from exposure play key, non-linear roles in determining the potential for detection of HGT. Sample size *n* is important, but frequently the practical sample sizes to be obtained are many orders of magnitude below the extant population size. It is therefore essential to wait until natural selection has had time to operate, to have any chance of effectively detecting horizontal gene transfer events.

A time lag between initial occurrence of rare HGTs and the opportunity of detection will therefore be present in most environments even though the relevant HGT events lead to positive selection of **transformant** bacteria (Nielsen and Townsend, 2001, 2004). Quantifying this time lag and determining the relationship between HGT frequencies and probability of detection requires the application of mathematical models with dependency on several key parameters: HGT frequencies, changes in relative fitness of the transformants, bacterial population sizes, and generation times in nature (Figure 1 and Box 1). A few studies have accordingly begun to characterize the effects of **natural selection** and the probability of **fixation** of HGT events in bacterial populations (Landis et al., 2000; Nielsen and Townsend, 2001, 2004; Johnson and Gerrish, 2002; Pettersen et al., 2005, Townsend et al., 2012). The multiple levels of, and importance of population genetic considerations in understanding HGT have recently also been reviewed by Baquero and Coque (2011 and references within) and Zur Wiesch et al. (2011).

KEY CONCEPT 3 | Transformant The individual cells in a larger bacterial population that have acquired DNA through horizontal gene transfer. The transformation frequency is often calculated as the number of transformant cells per the total number of recipient cells.

Box 1 **Key Model Equations for the Population Dynamics of HGT Events.**

Under fairly general assumptions, population genetic theory facilitates quantitative estimation of probabilities of population fixation of HGT events, as well as probabilities of detection of transfers that are en route to fixation. The first concept to undertake in consideration of these probabilities is the number of HGT events that occur over time *t*. Assuming that transfers are independent from one another and that the product of the exposed population size *n* and rate of transfer *r* is low,

(1) $$\mathrm{Pr}\{k\text{ actual transfers}\}=\frac{{\left(xrt\right)}^{k}{\text{e}}^{-nrt}}{k!}.$$

Nielsen and Townsend (2001), where Pr{} denotes “the probability of.” Assuming a standard decay rate λ for recombinogenic DNA material (a transgene), and a decay function for the population exposed over time of of *x*(*t*) = *x*_0_e^−λ*t*^, the

(2) $$\mathrm{Pr}\{\text{neutral transgene fixation}\}=\frac{x_{0}r}{N\lambda }.$$

Assuming large population size *N*, small selection coefficient *s*, and no interference between multiple positively selected alleles in the population,

(3) $$\mathrm{Pr}\{\text{selected transgenefixation}\}=\frac{2x_{0}rm}{\lambda }$$

(Nielsen and Townsend, 2001). In most practical contexts, HGT events will not have fixed throughout a population during the exposure period and time and time scale studied. Bacterial growth and competition may be modeled as in Hartl and Clark (1997) to yield the recombined DNA frequency *p* over time *t* after transfer based on a Malthusian selection coefficient *m*:

(4) $$p=\frac{{\text{e}}^{mt}}{N-1+{\text{e}}^{mt}}$$

(Nielsen and Townsend, 2004).

Applying Equation 1 and Kimura's diffusion equation result for the spread of a positively selected allele, it is further possible to integrate over all possible timings of potential HGT, from time *t* of zero, through time *t_s_* over which selection occurs, to time *t_x_* of sampling, to calculate in a given scenario that the

(5) $$\text{Pr}\{\text{transgene detected}\}=\left(\frac{1-e^{-2m}}{1-e^{-2Nm}}\right)rx\int_{0}^{t_{x}}\left(1-{\left(1-\frac{e^{m\left(t_{s}-t\right)}}{N-1+e^{m\left(t_{s}-t\right)}}\right)}^{n}\right)e^{-\left(\frac{1-e^{-2m}}{1-e^{-2Nm}}\right)rxt}dt$$

(Townsend et al., 2012). The primary experimental design criterion, however, would be the restricted, higher probability of detection given that a successful HGT has occurred; and frequently *t_x_* = *t_s_*. Provided sufficiently large *N* compared to *m*, then, a simpler result obtains:

(6) $$\text{Pr}\{\text{HGT detected given that it occurred}\}=1-e^{-(1-e^{-2m})rxt_{x}}.$$

KEY CONCEPT 4 | Natural selection The process by which traits become either more or less common in a population as a function of their heritable effect on reproductive success.

KEY CONCEPT 5 | Fixation A particular allele/gene is present in all members of the population.

In the recent Frontiers publication, Townsend et al. (2012), we integrated previous theory into a cohesive probabilistic framework to address current methodological shortcomings in the detection of HGT events. We assessed the key parameters and how they interact in a 5-dimensional graphical space. We also probabilistically modeled the time lag between HGT occurrence and detectability, accounting for the stochastic timing of rare HGT events; exploring also scenarios where bacteria are exposed to relevant DNA sources for a relatively short time. Our analysis yielded a simple formulation for the probability of detection given that a HGT actually occurred. This formulation facilitates computation of the statistical power of an experimental sampling design. Here we review key parameters determining the fate of HGT events in bacterial populations, consider complexity and uncertainty in parameter estimation, and identify knowledge gaps for further research.

## Population genetic parameters determining the outcome of HGT events

Within the population genetic framework, the key parameters determining the fate of rare HGT events in larger bacterial populations are

(i) The rate of HGT (*r*),
(iia) The bacterial population size (*N*),
(iiib) The fraction (*x*) of the bacterial population that is exposed to the relevant DNA,
(iii) The strength of selection on transformants (*m*), and
(iv) The bacterial generation time (*t*).

All these parameters are subject to considerable measurement uncertainty. Moreover, they are impacted by natural variability in biological systems and tempo-spatial interdependencies. Therefore, quantitative estimates of such parameters are best presented by ranges or probabilistic distributions, rather than point values. Measurement uncertainty can be reduced by proper statistical analysis and methodological design. Accounting for uncertainty in parameter estimation due to natural variability requires a much larger amount of data collection. Accounting for structural uncertainty due to insufficient knowledge of the broader biological system including implicit assumptions made on the nature of the HGT models applied can be acknowledged but requires extensive intense investigation of the system. In the absence of long-term basic research programs is challenging to address quantitatively. Below we consider the basis for obtaining quantitative parameter ranges that are informative in modeling approaches, and discuss their robustness and dependency on the model system in which they were generated.

### Rates of HGT

Several methodological approaches have been used to estimate or quantify HGT rates in bacterial populations. These methods have different limitations and advantages as discussed below.

#### Bioinformatics-based quantification

Bacterial genome sequencing has lead to major changes in the appreciation of the importance of HGT in bacterial evolution and adaptation (Ochman et al., 2000; Posada et al., 2002; Welch et al., 2002; Pal et al., 2005); although there is no well-established standard of proof, such studies frequently identify HGT events. Bacterial populations may also differ in their degree of accumulating HGT events (Boucher and Bapteste, 2009). For instance, bioinformatics-based genome comparisons by Dagan and Martin (2007), Dagan et al. (2008) suggest that up to 80% of the genes in proteobacterial genomes have been involved in lateral gene transfer. Due to constraints on representative sampling and sample size, genomics-based studies are often limited to identifying HGT events that have disseminated and persisted in the larger proportion of bacterial populations over relatively long time scales. Recently, the reduction in genome sequencing costs now enables the identification of HGT events taking place in metapopulations (Whitaker and Banfield, 2006; Forsberg et al., 2012) and sampled populations of related bacteria over shorter time scales (Hiller et al., 2010; Harris et al., 2012; Sousa and Hey, 2013). Despite their clear potential to illustrate the role of HGT in evolution of bacteria, these comparative genome analyses provide fundamentally imprecise quantifications of HGT rates. This imprecision arises because such analyses *de facto* examine the evolutionary successful progeny of the bacteria in which the HGT events occurred. Any direct numeric quantification of the HGT rate from comparative genomics data is therefore challenged by a lack of distinction between the rate that HGT events occur vs. the frequency that population dynamics spreads those events throughout species. Equivalently, comparative genomic analyses alone provide no information on the exposure rate of the sequenced genome to the same DNA fragment prior to the successful integration event, or the relative number of bacteria carrying the same HGT event that escape detection or have gone extinct during population growth (Pettersen et al., 2005). Finally, comparative genomic identification of HGT events rarely provides a precise identification of the time and location where the HGT event(s) occurred, the dependency on the specific donor DNA source, and the HGT vectors involved. These weaknesses arise inevitably from any approach relying on the analysis of single individuals sampled from an evolved and disseminated population of descendants of the primary transformants.

In summary, comparative genomic analyses, including metagenomics-based approaches, have been essential to elucidating the qualitative impact and evolutionary importance of HGT in bacterial populations. Such analyses do not yet, however, provide a quantitative understanding of HGT rates as they occur on a contemporary time scale.

#### Experimental-based quantification

The determination of HGT rates in a contemporary perspective is usually based on data obtained from environmental and laboratory-based experimental studies. These experimental studies can quantify HGT rates of DNA fragments of known composition in the model system used, as well as explore their dependency on experimental variables (Nordgård et al., 2007). Limitations to such studies arise from the biological model system considered, the choice and growth characteristics of the bacterial populations studied, from scaling issues and the limited capacity for analysis of individual cells in larger bacterial sample sizes. For instance, both experimentally-based laboratory studies as well as environmental sampling-based studies typically have a detection limit of 1 HGT event per 10^9^–10^10^ bacteria exposed (Nielsen and Townsend, 2004). Moreover, the precision of laboratory estimates for purposes of understanding processes in natural environments is compromised because the relevant environmental variables influencing HGT rates could easily be missed or misrepresented in many, most, or all model systems.

A frequent limitation to quantitative measurement of HGT rates is the narrow focus on the transferability of a particular DNA sequence into an introduced single recipient species over a limited time frame. In particular, studies of recombination with chromosomal DNA at predefined loci in bacteria suggest a log-linear relationship with increasing sequence divergence. Drawing general conclusions on HGT rates based on experimental HGT studies that have characterized the transferability and rate of a particular, predefined gene/locus (Zawadzki et al., 1995; Vulic et al., 1997; Majewski et al., 2000; Costechareyre et al., 2009; Ray et al., 2009) is, however, problematic. For instance, HGT rates established for a particular locus and donor/recipient pair may not necessarily represent the relative gene transfer potential for that locus into a broader set of bacterial recipients. Retchless and Lawrence (2007) showed by bioinformatics-based analyses that different parts of a bacterial genome have a non-uniform likelihood of recombination. Accordingly, Ray et al. (2009) demonstrated experimentally a high variability in gene transfer frequencies for the same donor/recipient pair depending on the genomic location of the transferred gene. The initial gene transfer frequencies that are determined between species will quantify only one aspect of the broader dynamics of the gene transfer process, as subsequent spread of the acquired DNA within a bacterial population can occur at several order of magnitude higher frequencies within species (Domingues et al., 2012a). Moreover, different members of the same bacterial population can also, due to random single mutations, have different HGT rates with divergent DNA (Rayssiguier et al., 1989; Matic et al., 1997; Townsend et al., 2003). The presence of mobile genetic elements may also increase local DNA sequence similarity between otherwise unrelated bacterial species, thereby providing opportunities for gene exchange through homologous recombination (Bensasson et al., 2004; Domingues et al., 2012a,b). Thus, the genomic location in the donor and the nature of the recombination site in the recipient cell, as well as the recombinogenic characteristics of single cells within the larger bacterial population will determine the transfer rates for a particular locus. This one example of the complexity of HGT illustrates the challenges in obtaining robust estimates of the HGT rates for a particular gene within or into a bacterial population.

To address this issue (in part), genome-wide studies of the recipients in HGT studies in laboratory populations are now emerging (Mell et al., 2011; Sauerbier et al., 2012). These latter studies suggest that multiple gene transfer events into the recipient genome often occur simultaneously after exposure to homologous genomic DNA. The outcome typically appears to be bacterial transformants with a variable proportion of between 1 and 3% of their genome carrying recombined regions. A recent study by Overballe-Petersen et al. (2013) suggest bacterial uptake of highly fragmented DNA can result in single HGT events that are producing as few as a single nucleotide change, an outcome rarely tested in previous experimental studies. Taken together, there is experimental evidence that a broad size range of DNA fragments can be acquired through HGT and that experimental data are available mostly for a subset of these size-ranges.

In summary, the quantification of HGT rates between bacterial genomes will vary with the locus examined and the model system used including the choice of donor/recipient pair as well as experimental variables. Considerations of HGT rates in bacterial populations should take into account that initially rare interspecies HGT events may subsequently transfer at high frequencies among members of the same population (Novozhilov et al., 2005; Domingues et al., 2012a). Theory (Jain et al., 1999) as well as recent, locus-context dependent or locus independent transfer models suggest more complex patterns of HGT. Such studies also suggest that robust estimates of HGT rates cannot currently be obtained in experimental models that will reflect transfer rates as they occur in complex natural systems. Nevertheless, laboratory studies are frequently viewed as representing upper levels of HGT rates for a given donor-recipient combination, and can therefore be useful for limiting the parameter ranges considered.

#### Sampling-based detection

Taking into account the complexity of the HGT process, experimental models will only rarely reproduce environmental conditions that can reflect HGT rates as they occur naturally. In the face of this complexity, more precise quantitative estimates of HGT rates can conceivably be obtained by the empirical approach of sampling native bacterial populations exposed to defined DNA sources. Such sampling can circumvent several of the methodological limitations of laboratory-based gene transfer models. Sampling and characterization of environmental populations of bacterial cells and populations for HGT, however, presents their own challenges (Nielsen and Townsend, 2004). A restricted sampling capacity of large populations results in an inevitably low power. In heterogeneous environments, the ability to examine relevant recipient species populations for specific HGT events is limited due to methodological constraints related to cultivation and genetic analysis. Current methodologies are mostly limited to the genetic analysis of approx. 10^3^–10^5^ single bacterial cells. Recent HGT events occurring at low frequencies may be nearly impossible to detect in populations that are much larger than this.

In summary, quantification of HGT rates by sampling natural bacterial populations is feasible but restricted by practical methodological constraints. The quantification of HGT events as they occur under natural conditions undoubtedly provides the better estimate of HGT rates used for modeling approaches. Only a few studies are however available on HGT rates based on sampling based approaches. Both experimental and natural sampling-based approaches carry important assumptions on HGT rates, and in both cases the relevant rates can be measured only within a limited sampling period that undoubtedly cannot be comprehensive of all potentially relevant environmental conditions.

### Bacterial population size

Bacterial populations often fluctuate in size reflecting highly opportunistic population dynamics. Sizes depend on habitat and resource availability. In the gastrointestinal tract of mammals, population sizes are typically in the range of 10^6^–10^9^, and in soil, 10^7^–10^9^ per gram of material. Quantification of bacterial population sizes can be challenging as often only a small fraction of bacteria is amendable to cultivation (e.g., in soil or the gastrointestinal tract; Janssen et al., 2002; McNamara et al., 2002; Zoetendal et al., 2004). Moreover, the ability to culture bacteria (including those in a viable but non-culturable state) will vary with environmental conditions, resulting in major methodological challenges to the culture-based determination of the population sizes for bacterial species in their communities and environments.

Additionally, fluctuating populations of bacteria are usually not evenly distributed in a given environment (Bulgarelli et al., 2012). Bacterial populations form local aggregates, clusters, and biofilms that can be isolated, partly isolated, or in contact with one another. For instance bacteria on leaf surface forms spatially structured aggregates (Kinkel et al., 1995; Morris et al., 1997; Monier and Lindow, 2004). Incorporating both the spatially structured dispersion and population fluctuations, bacterial species may best be described as **metapopulations** (Walters et al., 2012; Fren et al., 2013). Metapopulations in close proximity are more likely to share genetic material than more distant populations (Cadillo-Quiroz et al., 2012; Polz et al., 2013). Moreover, strongly positively selected traits (and phenotypes) are likely to survive and expand locally before further dissemination to more distant populations. Such dynamics call for careful consideration in terms of constructing a proper sampling design.

KEY CONCEPT 6 | Metapopulation A group of spatially separated populations of the same species that nonetheless have interactions, maintaining a species identity.

For bacterial populations, the spatial aspects engendered by bacterial growth dynamics are relevant on diverse scales. For example, in a soil environment, some bacteria will be found in the immediate proximity of the plant roots, and are likely to be exposed to root exudates. Other bacteria, just a few millimeters away will not accrue benefit from the same nutrient source, must compete for sparser resources, and hence exhibit smaller population sizes and slower growth rates. On a larger scale, the root systems of individual plants may be seen as discrete patches, islands or sources, but with a potential for microbial dispersal between. Topographically or ecologically demarcated fields or plots represent another level of scale, with different modes and vectors for potential transfer (dispersal) in between. Spatially structured population models have not yet been incorporated in modeling of HGT (Townsend et al., 2012). Incorporation of spatial structuring and heterogeneity is likely to be an important next step for quantitative modeling, as it will affect the population dynamics of rare bacterial phenotypes in larger populations (Fren et al., 2013). An accurate determination of *N* is also challenged by the lack of a precise understanding of coherent bacterial populations and the species definition in prokaryotes (Cohan, 2002, 2005; Fraser et al., 2007, 2009; Achtman and Wagner, 2008).

In summary, bacterial populations vary in size and structure within the environment. This variation will have implications for the likelihood of occurrence as well as the subsequent population dynamics of HGT events. While quantitative estimates of bacterial population sizes (*N*) can usually be obtained for the biological system of interest, it is challenging for such estimates to accurately account for the local fluctuations and patterns of bacterial distribution, and differences between relevant bacterial phenotypes (species).

### Fraction of bacterial population exposed to DNA

Given a spatially structured population, DNA exposure will be uneven across the recipient population; the distribution of recipients is expected to be dissimilar from the distribution of the DNA source. These differences in exposure make accurate modeling challenging as well as muddling empirical efforts to parameterize a model with the fraction of bacteria in a population that may undergo HGT as a result of exposure to a given DNA source. The presence of physical and biotic barriers to DNA exposure in most environments suggests that the fraction of potential recipients exposed would inevitably be considerably less than one. Empirical data or experimental models that permit quantification of actual DNA exposure levels in microbial communities are typically not available. DNA exposure will also depend on the biological and physical properties of the DNA vector or DNA source. An upper bound for exposure can be determined if the absolute concentration (or copy number) and the dynamics or decay rate of the particular DNA source in question is known. Nevertheless, parameter estimates of the fraction of the bacterial population (*n*) exposed to the DNA source will be based on theoretical considerations relying on the known properties of the DNA source and recipient population.

### Strength of selection

The detection of rare HGT events in larger populations is typically feasible only if the few initial transformants have a growth advantage, so that they increase their relative proportion in the overall population. Selection in microbial populations is typically described by the Malthusian fitness parameter *m*, which works out to be equal to the selection coefficient in haploids (Hartl and Clark, 1997). The parameter *m* represents the relative cost or advantage conferred by the HGT event to the transformed bacterium compared to untransformed members of the same population (*m* = 0 represents no positive or negative selection). In nature, values of *m* that are relevant to HGT success would range from very weak positive selection (the reciprocal of population size, perhaps *m* = 10^−12^) to strong positive selection (perhaps at most *m* = 1, representing a doubling of the rate of reproduction).

In certain circumstances, *m* could be greater than one in relative terms: stronger positive selection would, for instance, arise under intense antibiotic treatment when the acquisition of a resistance gene is exceptionally advantageous, such as when 100% of the susceptible population is likely to die. Thus, the relative growth advantage can be immense, leading to rapid population expansion and replacement of the transformant population in the absence of competitors. Clinical antibiotic usage produces strong fluctuations in the selection for a given resistance trait over time. Such exceptionally strong periodic selection is not considered further here and would require different modeling approaches such as **dynamic epidemiological modeling**. In opposite cases, horizontally acquired DNA may be costly (Baltrus, 2013) or outright lethal to the recipient cell (Sorek et al., 2007). In most cases, these two extremes are unexpected. For most relevant HGT events and acquired traits, values of *m* of low magnitude are expected, and constant values of *m* are assumed over time (Townsend et al., 2012). These typical, low values of *m* will not correspond to levels of selection that are easily discriminated in the laboratory populations of bacteria over limited time periods, but will affect relative growth in nature over time.

KEY CONCEPT 7 | Dynamic epidemiological modeling The use of mathematical models to project how infectious diseases progress and to predict the outcomes of interventions.

Quantification of the strength of selection is affected by model inconstancy. The selection coefficient of a given trait is typically *not* constant over evolutionary time but will fluctuate over space and time, responding to environmental variables, varying among bacterial genotypes, and showing gene-by-environment interactions (Kimura, 1954; Barker and Butcher, 1966; Via and Lande, 1985, 1987; Hodgins-Davis and Townsend, 2009). For instance, an antibiotic resistance trait can be highly advantageous in the presence of antibiotics but confer a fatal fitness cost in the absence of antibiotics (Johnsen et al., 2009, 2011). Thus, a numerically constant selection coefficient will by nature be an inexact approximation and careful consideration is required for quantification over variable environments. In addition to environmental fluctuations, the genome of a given bacterial transformant is not constant. Further HGT events and continual mutational processes may rapidly change the initial effects on host fitness of a given HGT event (Lenski et al., 1991; Gerrish, 2001; Heffernan and Wahl, 2002; Rozen et al., 2002; Barrett et al., 2006; Starikova et al., 2012). For instance, biological constraint to functional HGT events as represented by unconstrained gene expression or differences in codon usage patterns may be ameliorated over time through spontaneous mutations (Lawrence and Ochman, 1997; Tuller et al., 2011; Starikova et al., 2012).

In summary, the strength of selection for a given trait depends on host genetics and environmental conditions. Natural variability and complexity in such systems obstruct precise quantification of selection. Similar to HGT rates, selection is better expressed with a value range and modeled as multiple scenarios using long-term average values. Experimental models, in particular, will rarely provide a precise estimate of the selection coefficient under natural conditions, and have potential only to be able to identify relatively strong selection coefficients. Acknowledging the limitations to quantification, mathematical models should incorporate uncertainty in the estimation of the strength of selection and examine a broad parameter range of *m*.

### Bacterial generation time

Bacterial cell division time varies with species and environments (Powell, 1956; Kovarova-Kovar and Egli, 1998). Division time can be as short as <1 h in nutrient-rich environments with stable temperatures such as the gastrointestinal tract, and can be as long as several weeks in nutrient-limited and temperature-fluctuating environments such as soil. Bacterial populations with spore-forming capacity or with “persister” stages (Dawson et al., 2011) lead to non-uniform duplication rates among cells present in a population. Variable nutrient access over time and space also lead to variation in generation time, even in homogeneous bacterial culture conditions (Kovarova-Kovar and Egli, 1998). Estimates of bacterial generation time should therefore be interpreted as average population measurements with potentially very large cell-to-cell differences. Quantitative values for bacterial generation time can be obtained for cultivable species though experimental measurements in the laboratory. Actual generation times of bacterial species, as measured in their natural environments, are only rarely available.

Bacterial generation time will also be determined by the overall bacterial population and community structure; particularly if opportunities for population expansion exist. The infectious lifestyle of some pathogens leads to exceptionally rapid changes in their population sizes and generation time (during infections) followed by strong **bottlenecks** (e.g., during transmission). Thus, depending on the pathogen in question, the transformed cells may or may not be competing for resources with non-transformed members of their populations. With the exception of some chronic bacterial infections, most changes in relative population sizes of pathogens are expected to take place through population replacement. The speed of replacement will be based on competitive advantage of the transformant population as expressed through the selection coefficient and observed through a more rapid generation time. Thus, the materialized growth advantage is observed in the presence of a non-transformed population.

KEY CONCEPT 8 | Bottleneck An event in which a population is reduced to a very small size, eliminating most genetic variation.

In summary, quantitative estimates of bacterial generation time (*t*) can be obtained for cultivable bacterial species. Large tempospatial variation is expected within individual members of a population and different lifestyles will determine the characteristics and limits to bacterial growth rates.

#### General considerations

Above we have described some key features of the parameters defining the fate of rare HGT events occurring in larger bacterial populations. These parameters have in common that they cannot be precisely quantified as a defined single numeric value for a given bacterial population and environment. Complex bacterial population dynamics and interactions lead to large temporal and spatial variability in parameter values in structured environments. This natural variability can nevertheless be captured in modeling approaches by determining the effect of a range or likely distribution of parameter values for a given environmental HGT scenario. Multiple outcomes/scenarios can be quantified for their probability and critical parameter ranges can be identified that can structure further hypothesis formulation, guide experimental design and contribute to theory development.

The utility of a quantitative approach to understand HGT processes as presented in Townsend et al. (2012) is, although dependent on some knowledge of the rates of the relevant processes, independent of the specific bacterial mechanism of HGT (e.g., transduction, conjugation, transformation). Quantitative adjustments to the DNA exposure and HGT rate can in our model accommodate diverse mechanisms of transfer of non-mobile DNA. Other models have been developed to better understand how mobile genetic elements can move within and between bacterial populations (e.g., Bergstrom et al., 2000; Novozhilov et al., 2005; Ponciano et al., 2005). Although out of the scope of this review, information from both types of models may be needed to generate a more complete picture of HGT in bacteria. Figure 1 illustrates the key parameters of a quantitative approach.

An outcome of the quantitative analysis is that measured HGT frequencies are typically highly insufficient as predictors of the short and long term evolutionary impact of HGT events. As long as such events occur repeatedly, other factors will determine the biological impact of these events (Pettersen et al., 2005). Directional selection typically dominates determination of the probability of detection of HGT events (Townsend et al., 2012). Strong experimental sampling designs would therefore avoid overly focusing on the possibility of detection of the initial HGT *recipients* (and associated HGT frequencies), but rather attempt to capture the population dynamics of positively selected *descendants* of the primary transformants. Generally, experimental design in this context will incorporate a delay between exposure and assessment, facilitating the action of selection to bring descendants of recipients to higher, detectable frequency. Experimental designs should explicitly address the intensity of selection that they should be able to detect given the design, just as (for example) clinical trial designs specify an effect size for a treatment that they should be able to detect.

Accordingly, a key consideration is when the transformant proportion rises to the point where subsequent evolution is largely deterministic based on the current level of directional selection (Rouzine et al., 2001). Understanding threshold levels of transformant populations also have practical implications. For instance, the prevalence of a pathogenic strain carrying an HGT event encoding antibiotic resistance for first line antibiotic therapy is of highest interest when its relative proportion among sensitive strains has reached <0.1–0.3, as such levels will call for changes in clinical prescription guidance (Daneman et al., 2008).

Even when bacteria experience positive selection, stochastic processes contribute to variability in the predicted fate of nearly neutral and weakly selected transformants. **Genetic drift**, uneven survival rates due to bottlenecks and **selective sweeps** in structured bacterial populations can also play important roles in determining the fate of individual genotypes in larger populations (Majewski and Cohan, 1999; Heffernan and Wahl, 2002; Pettersen et al., 2005). Random or seasonal variations in local population sizes may also cause particular genotypes (e.g., transformants and their descendants) to fluctuate at low frequencies above or below detection for long periods of time in spatially structured populations (Gerrish and Lenski, 1998).

KEY CONCEPT 9 | Genetic drift The counterpart of natural selection, genetic drift is change in the frequency of a gene variant within a population due to “random sampling”–the chance association of the variant with individuals of differing reproductive success.

KEY CONCEPT 10 | Selective sweep An event in which a highly selected genetic variant confers a large reproductive advantage and carries with it only the linked genetic variants, sweeping other variation out of a population.

Spatially structured models have not yet been incorporated in modeling of HGT (Townsend et al., 2012) and are likely to be an important next step for quantitative assessment and modeling. Experimental designs should accommodate the potential for rare HGT events to be unevenly distributed in large, structured bacterial populations. Bacterial species are expected to have non-uniform distribution of genes at spatial and temporal scales (Reno et al., 2009). For instance, antibiotic resistance genes or transgenes are likely to be initially present only in a limited number of patches (e.g., patients/hospitals, or soil sites/fields), representing sub- or meta-populations of the larger global population (Maynard Smith, 2000). The fate of initial HGT events established in some metapopulations will depend on migration or dispersal for further dissemination in the larger bacterial population. Bacterial dispersal relies on a multitude of factors and is expected to be of variable intensity and directionality. Bacterial dispersal can be dependent on or at least enhanced by vectors: for antibiotic-resistant bacteria, humans have been excellent vectors, carrying bacteria and their transferable traits between hospitals across continents. In soil, numerous small and large invertebrates may carry soil and bacteria around. At larger scales, water, wind, animals, birds, food and feed products, and human activities carry and disseminate bacteria both locally and globally.

Understanding the relationship between the exposed and total population size, HGT rates, bacterial generation time, selective advantage, and delayed sampling, is not an easy task. Nevertheless, it is essential to the fate of HGT events occurring in various environments. The model published by Townsend et al. (2012) examines how these parameters define the outcome of the fate of HGT occurring in various environmental scenarios. It was concluded that under some conditions HGT is likely to occur over temporal and spatial scales that are not amenable to direct experimental observation; emphasizing that the probability of detection can only correspond to a calculable level of selection, and that a powerful experimental design requires a delayed sampling strategy (i.e., not close to the initial exposure and the HGT event itself). Greater informed quantitative analysis of the population genetics of the bacterial system(s) investigated will contribute to make the assumptions behind hypothesis formulation less arbitrary and more explicit; and therefore, improve the robustness of future experimental designs.

### Conflict of interest statement

The authors declare that the research was conducted in the absence of any commercial or financial relationships that could be construed as a potential conflict of interest.
